# Case report: Long-term follow-up of a large full-thickness skin defect treated with a photosynthetic scaffold for dermal regeneration

**DOI:** 10.3389/fbioe.2022.1004155

**Published:** 2022-12-01

**Authors:** Miguel L. Obaíd, Felipe Carvajal, Juan Pablo Camacho, Rocío Corrales-Orovio, Ximena Martorell, Juan Varas, Wilfredo Calderón, Christian Dani Guzmán, Marianne Brenet, Margarita Castro, Cecilia Orlandi, Sebastián San Martín, Antonio Eblen-Zajjur, José Tomás Egaña

**Affiliations:** ^1^ Department of Plastic Surgery, Hospital del Salvador, Santiago, Chile; ^2^ Institute for Biological and Medical Engineering, Schools of Engineering, Medicine and Biological Sciences, Pontificia Universidad Católica de Chile, Santiago, Chile; ^3^ Division of Hand, Plastic and Aesthetic Surgery, University Hospital, LMU, Munich, Germany; ^4^ Critical Care Unit, Hospital del Salvador, Santiago, Chile; ^5^ Biomedical Research Center, School of Medicine, Universidad de Valparaíso, Valparaíso, Chile; ^6^ Sky-Walkers SpA, Litueche, Chile; ^7^ Clínica Orlandi Dermatological Center, Santiago, Chile; ^8^ Translational Neuroscience Lab, Faculty of Medicine, Universidad Diego Portales, Santiago, Chile

**Keywords:** photosyntetic scaffold, wound regeneration, tissue engineering, photosynthetic biomaterial, long term treatment, case report

## Abstract

It is broadly described that almost every step of the regeneration process requires proper levels of oxygen supply; however, due to the vascular disruption in wounds, oxygen availability is reduced, being detrimental to the regeneration process. Therefore, the development of novel biomaterials combined with improved clinical procedures to promote wound oxygenation is an active field of research in regenerative medicine. This case report derives from a cohort of patients enrolled in a previously published ongoing phase I clinical trial (NCT03960164), to assess safety of photosynthetic scaffolds for the treatment of full skin defects. Here, we present a 56 year old patient, with a scar contracture in the cubital fossa, which impaired the elbow extension significantly affecting her quality of life. As part of the treatment, the scar contracture was removed, and the full-thickness wound generated was surgically covered with a photosynthetic scaffold for dermal regeneration, which was illuminated to promote local oxygen production. Then, in a second procedure, an autograft was implanted on top of the scaffold and the patient’s progress was followed for up to 17 months. Successful outcome of the whole procedure was measured as improvement in functionality, clinical appearance, and self-perception of the treated area. This case report underscores the long-term safety and applicability of photosynthetic scaffolds for dermal regeneration and their stable compatibility with other surgical procedures such as autograft application. Moreover, this report also shows the ability to further improve the clinical outcome of this procedure by means of dermal vacuum massage therapy and, more importantly, shows an overall long-term improvement in patient´s quality of life, supporting the translation of photosynthetic therapies into human patients.

## Introduction

Wound healing and tissue regeneration is a highly complex process that requires the orchestration of several physiological and molecular processes (Martin and Nunan, 2015). This may result in tissue repair of diverse quality, ranging from pathologic fibrosis to functional scarring (Rippa et al., 2019) and, therefore, the establishment of novel approaches for wound treatment is required, in order to improve the regeneration process to achieve a successful and efficient repair of the damaged tissue. Within this context, the clinical translation of novel biomaterials and procedures into clinical practice represents a key challenge in regenerative medicine, being an active field of research.

As tissue repair and regeneration rely in the presence of several bioactive molecules, novel approaches for the local and controlled released of therapeutic agents have been proposed within the last decade, including the delivery of growth factors (Jarquín-Cordero et al., 2020), immunomodulatory agents (Kharaziha et al., 2021), antibiotics (Shi et al., 2021) and oxygen (Dissemond et al., 2015). This last molecule has been broadly described to play a critical role in wound healing, and the lack of proper oxygen supply is related with poor clinical outcome and wound chronicity (Rodriguez et al., 2008). Among others, the use of chemical and mechanical (Dissemond et al., 2015) methods for oxygen delivery have been evaluated in clinical settings but, to date, no conclusive results have been described.

More recently, the use of photosynthetic microorganisms have been proposed as an alternative way for tissue oxygenation (Chávez et al., 2020; Wang et al., 2021), showing promising results in several *in vitro* and *in vivo* models, including tumor treatment (Huo et al., 2020), myocardial ischemia (Cohen et al., 2017), organ preservation (Veloso-Giménez et al., 2021) and tissue regeneration (Hopfner et al., 2014; Schenck et al., 2015; Chávez et al., 2016; Centeno-Cerdas et al., 2018).

Although results are promising for photosynthetic therapies, only one single clinical trial, conducted by our research group, has been reported showing the safety of this approach in humans (Obaíd et al., 2021), but no long-term results have been described so far. The presented clinical case, derived from the aforementioned clinical trial aims at determining the long-term effects of implanting photosynthetic cells in humans, particularly in the process of wound healing. Hence, here we present the first case report of a 17-months follow-up of a large full-thickness skin defect treated with photosynthetic scaffolds.

### Photosynthetic scaffold fabrication and implementation

The photosynthetic scaffold used in the treatment of the patient presented here, was manufactured according to the procedures described in (Obaíd et al., 2021). Briefly, moist 25 cm^2^ patches of Integra^®^ dermal regeneration template (DRT) were seeded with 1.25 × 10 ^8^ cells of *Chlamydomonas reinhardtii* microalgae, embedded in Evicel^®^ human fibrin sealant, and left to grow for 4 days under constant illumination. After microbiology tests, to ensure the absence of pathogens, the photosynthetic scaffolds were taken to the operation room for their surgical implantation.

After implantation, the area was illuminated with a blue LED illumination device fabricated for use in the clinical trial, to stimulate photosynthesis for 7 days. After this time, the treated area was clinically monitored, and conventional dressings replaced periodically until the autograft surgery (Supplementary Figure S1).

## Case description

This case report describes a detailed surgical procedure and extensive follow-up of one of the first patients to receive a photosynthetic scaffold for the treatment of a full-thickness skin defect. This patient is part of a previously published cohort (Obaíd et al., 2021) of an ongoing phase-1 clinical trial (NCT03960164).

Briefly, human patients between 18–65 years, with non-infected acute full-thickness wounds and homogeneous granulation bed were selected for participation in the study. Patients with autoimmune diseases, immunosuppression treatments, psychiatric disorders and wounds located in the facial area were excluded. General treatment consisted in two surgeries where a photosynthetic scaffold was first implanted and cover with an illumination device for 7 days. Afterwards, a second surgery was performed to cover the regenerating tissue with an autologous skin graft. After a 90 days follow up, overall results showed that the presence of photosynthetic microalgae did not trigger deleterious reactions in the eight treated patients, allowing successful tissue regeneration. The case report presented here corresponds to patient 2 of aforementioned clinical trial (Obaíd et al., 2021). The present case corresponds to a 56-year-old female patient with a history of scald in childhood at the right arm. The main sequel included a scar contracture widely extended in the elbow flexion area including the cubital fossa. Functional limitation, especially for elbow extension, was exacerbated due to body growth, where the scar maintained its original size, while surrounding skin was growing and developing until adulthood. The fibrotic scar tissue presented complete loss of elasticity, with sensory, proprioceptive, autonomic and pain alterations, limiting the elbow flexion arc by 20° from full extension, therefore affecting normal maneuvers and impacting her quality of life. The scar was 11 cm long and 9 cm wide (∼80 cm^2^), and the local mechanical tension induced a chronic and painful ulcer in its center, with exacerbation of this lesion each time the patient tried to extend her arm. She never received other treatment or surgery at the scar site and did not present another relevant comorbidity. This patient was chosen for a long-term follow-up due to the extension of the treated wound, being the largest among the patients in the clinical trial, and because the outcome of the procedure can be functionally tracked, by studying the elbow extension capacity.

As part of the treatment presented here, two surgical procedures were performed. In the first one, the scar contracture was removed, generating a full-thickness skin defect located in the elbow flexion area, where the photosynthetic scaffold for dermal regeneration was implanted. Three weeks later, a split-thickness autograft was implanted on top of the scaffold.

After successful completion of both procedures, the patient evolution was monitored for up to 17 months, finishing her treatment and recovery with a vacuum massage therapy procedure, to finally discharge her with improved arm functionality and esthetics. This case corresponds to the largest defect treated among patients involved in the clinical trial, which led us to extensively report her condition and progress.

### Surgical procedures

**FIGURE 1:**
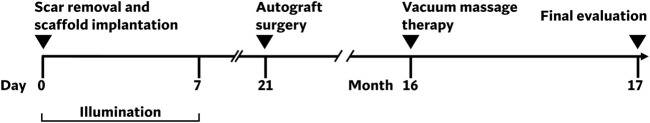
Timeline of the procedures. Scar was removed and the photosynthetic scaffold was implanted to cover the newly generated wound (day 0). During the first 7 days after surgery, implanted photosynthetic scaffold was illuminated. On day 21 after implantation, a split-thickness autograft was surgically fixed on top of the previously implanted photosynthetic scaffold. At month 16, a vacuum massage therapy was applied to the recovered area. Patient was monitored for a total of 17 months after the first surgery.

The complete procedure performed consisted of two surgeries: first, the scar was fully removed, and the generated wound was implanted with a scaffold for dermal regeneration loaded with oxygen-producing photosynthetic cells, i.e., a photosynthetic scaffold. After 21 days, a second surgery was carried out to implant an autograft on top of the photosynthetic scaffold. Both surgeries were performed under general anesthesia. Finally, to improve the functionality in terms of mechanical and biophysical characteristics of the skin graft, a vacuum massage therapy was performed. An overall timeline of the procedures is shown in Figure 1.

#### Scar removal and photosynthetic scaffold implantation

**FIGURE 2:**
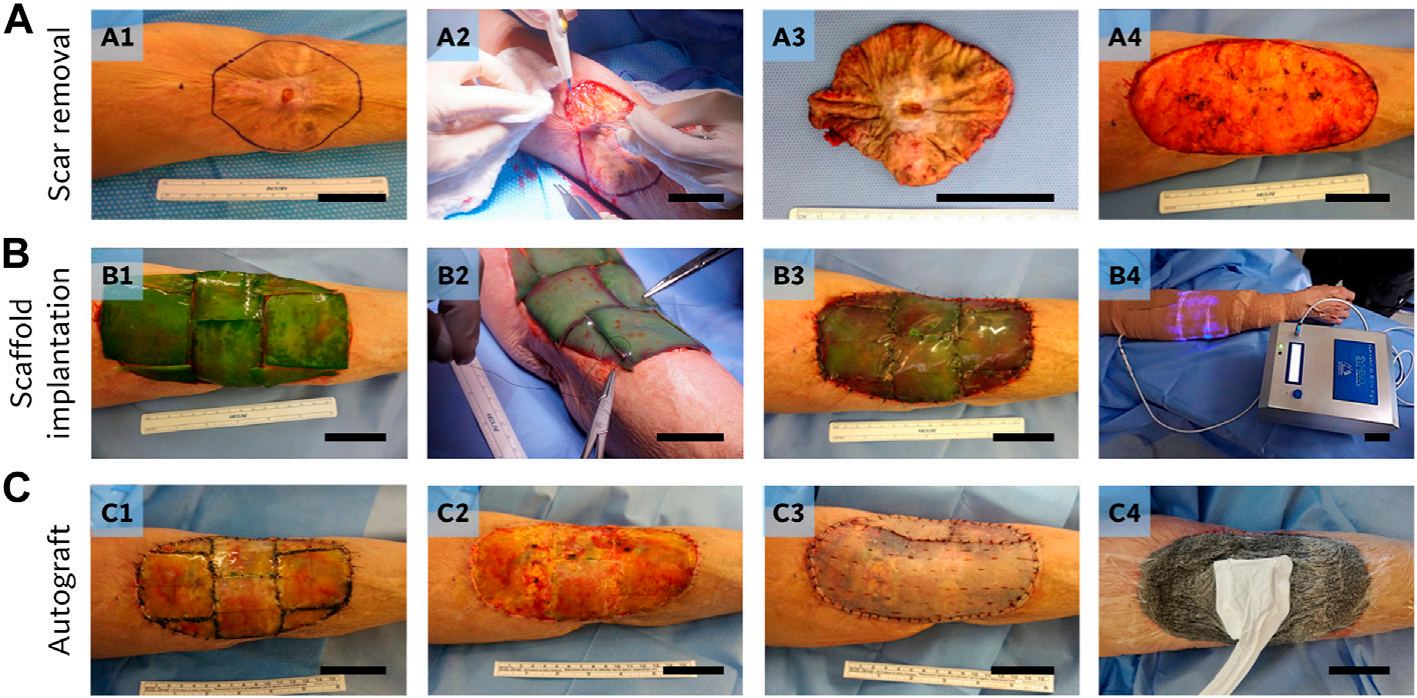
Surgical procedures. Scar removal **(A)**: edges were marked prior to resection (A1), and a complete scar excision was performed (A2-3), exposing the subcutaneous adipose tissue and the final size of the defect (A4). Scaffold implantation **(B)**: five sheets of photosynthetic scaffold (5 × 5 cm each) were required to cover the wound (B1), being cut-fitted, and fixed to the defect with non-absorbable sutures (B2-3). Finally, scaffolds were covered with a sterile dressing and the illumination device was placed on top (B4). Autograft **(C)**: 21 days after implantation, no signs of rejection, inflammation or infection were detected in the scaffolds, and a loss of the characteristic initial green color of the scaffold was observed (C1). After removal of the silicon layer (C2), an autograft was implanted (C3) and negative wound therapy was applied (C4). Scale bar represents 5 cm.

Complete excision of the burn scar contracture (Figure 2A1), including the central chronic ulcer, was performed with an electrosurgical unit, reaching the healthy adipose tissue of the subcutaneous plane (Figure 2A2). Detail of the removed burn scar tissue is shown in Figure 2A3. Due to the release of tension on the surrounding healthy skin, the size of the wound increased from 11 × 9 cm (∼80 cm^2^) to 18 × 10 cm (∼134 cm^2^), allowing a fully functional flexo-extension of the elbow (Figure 2A4). Photosynthetic scaffold implantation was performed as previously described (Obaíd et al., 2021). After complete removal of the scar, the wound bed was washed with sterile saline solution, while photosynthetic scaffolds were measured and cut to fit and cover the defect (Figure 2B1). Next, scaffolds were fixed with nylon 4/0 non-absorbable monofilament sutures (Ethilon^®^, Johnson&Johnson, NJ, USA) to the wound edges and between them, leaving the scaffold in direct contact with the wound bed (Figures 2B2,B3). The treated area was then covered with a transparent non-adherent contact dressing (Tegaderm^®^, 3M, MN, USA), followed by a thin absorbent cotton dressing. The illumination device (Sky-Walkers SpA, Litueche, Chile) was placed on top of the dressings and secured on site with an elastic bandage (Coban^®^, 3M, MN, USA). Finally, the illumination device was turned on (Figure 2B4) as previously described (Obaíd et al., 2021). Over the next 7 days, the patient’s elbow was kept in extension, by positioning the arm in an orthopedic device fixed to the arm and forearm with velcro straps, to avoid movement and friction in the intervened area. The patient remained hospitalized for 7 days, with daily control of vital signs, general clinical condition, and continuous monitoring of the implanted photosynthetic scaffold and the dressings by the clinical team. No clinical nor laboratory evidence of erythema, edema, inflammation, and infection was found during this postsurgical time. After 7 days, the illumination system was removed and patient was discharged from the hospital, receiving ambulatory follow-up and frequent replacement of wound dressings until the autograft surgery. Information regarding the patient’s perception of pain, itch, burning, smell, and light annoyance after the surgery is included in Supplementary Figure S2. Adequate integration of the photosynthetic scaffold to the initial wound bed was observed, allowing a complete elbow extension. The absence of signs of inflammation or infection in the surrounding healthy skin enabled the team to proceed with the skin autograft surgery at day 21 (Figures 2C1,C2). Clinical and laboratory follow-ups were complemented with wound biopsy of the implanted area, confirming the macroscopic clinical observations, with good integration of the scaffold to the patient’s tissue, and no findings regarding inflammation or signs of infection (Obaíd et al., 2021). Systemic response to the presence of the photosynthetic scaffold was also evaluated, by observing distribution of blood inflammation markers, which showed no systemic effect of the photosynthetic scaffold implantation (Obaíd et al., 2021).

#### Autograft surgery

Sutures and the silicone layer of the photosynthetic scaffold were removed, showing that the remaining collagen scaffold was correctly integrated to the wound bed (Figure 2C2). After washing with sterile saline solution, a 0.2 mm thick partial thickness skin autograft was implanted on top of the previously implanted scaffold (Figure 2C3). The autograft was obtained with a dermatome (Acculan^®^3Ti, Aesculap-BBraun, Tuttlingen, Germany) from the thigh of the patient. Small fenestrations were made to the skin graft with a scalpel to prevent the accumulation of exudate, and to ensure its correct adherence in the postoperative period. The graft was attached to the surrounding healthy skin with a non-absorbable nylon suture (Ethilon^®^, Johnson&Johnson, NJ, USA). Finally, a paraffin gauze dressing was overlaid, and a negative-pressure wound therapy dressing (Renasys^®^, Smith & Nephew, Hull, England) was positioned over the intervened area, which was maintained for 5 days at -120 mmHg (Figure 2C4).

### Nonsurgical procedures and functional characterization of the regenerated tissue

Sixteen months after the first surgery, the functionality of the intervened area was evaluated in terms of its barrier function as well as its mechanical properties. Moreover, the ability of the new tissue to respond to treatment was also studied. Non-invasive analysis of different skin parameters was performed in the Clinica Orlandi Dermatological Center (Santiago, Chile). Parameters, such as skin hydration, trans-epidermal water loss and skin flexibility were measured as widely described for other research works related to skin functionality and response to treatments (Krueger et al., 2011; Kim et al., 2013; Algiert-Zielińska et al., 2019; Sekine et al., 2020; Barrionuevo-Gonzalez et al., 2021; Chaiwong et al., 2022; Courage+Khazaka Electronic GmbH, 2022b; Courage+Khazaka Electronic GmbH., 2022a; Courage+Khazaka Electronic GmbH., 2022c). Skin hydration is measured instrumentally by a Corneometer^®^, based on the capacitance changes in the stratum corneum of the skin due to its water content. Trans-epidermal water loss is measured instrumentally by a Tewameter^®^, based on the water evaporation measured on a hollow cylindrical probe positioned over the skin. Skin flexibility is quantified using a Cutometer^®^, using a suction probe and measuring the skin deformation relative to the negative pressure applied.

All measurements were conducted under controlled ambient conditions, ensuring that the skin was clean and free of topical products, and the contralateral healthy cubital fossa was used as control of healthy tissue. After the first long term measurements, we decided to explore if further functional improvement of the treated area could be achieved by local vacuum therapy (Skintonic^®^ device, Skinexians, Pont-Eveque, France), a technique designed to stimulate local blood and lymphatic circulation.

Five ambulatory weekly sessions of vacuum therapy were carried out in the regenerated area and changes in key parameters were evaluated.

**FIGURE 3:**
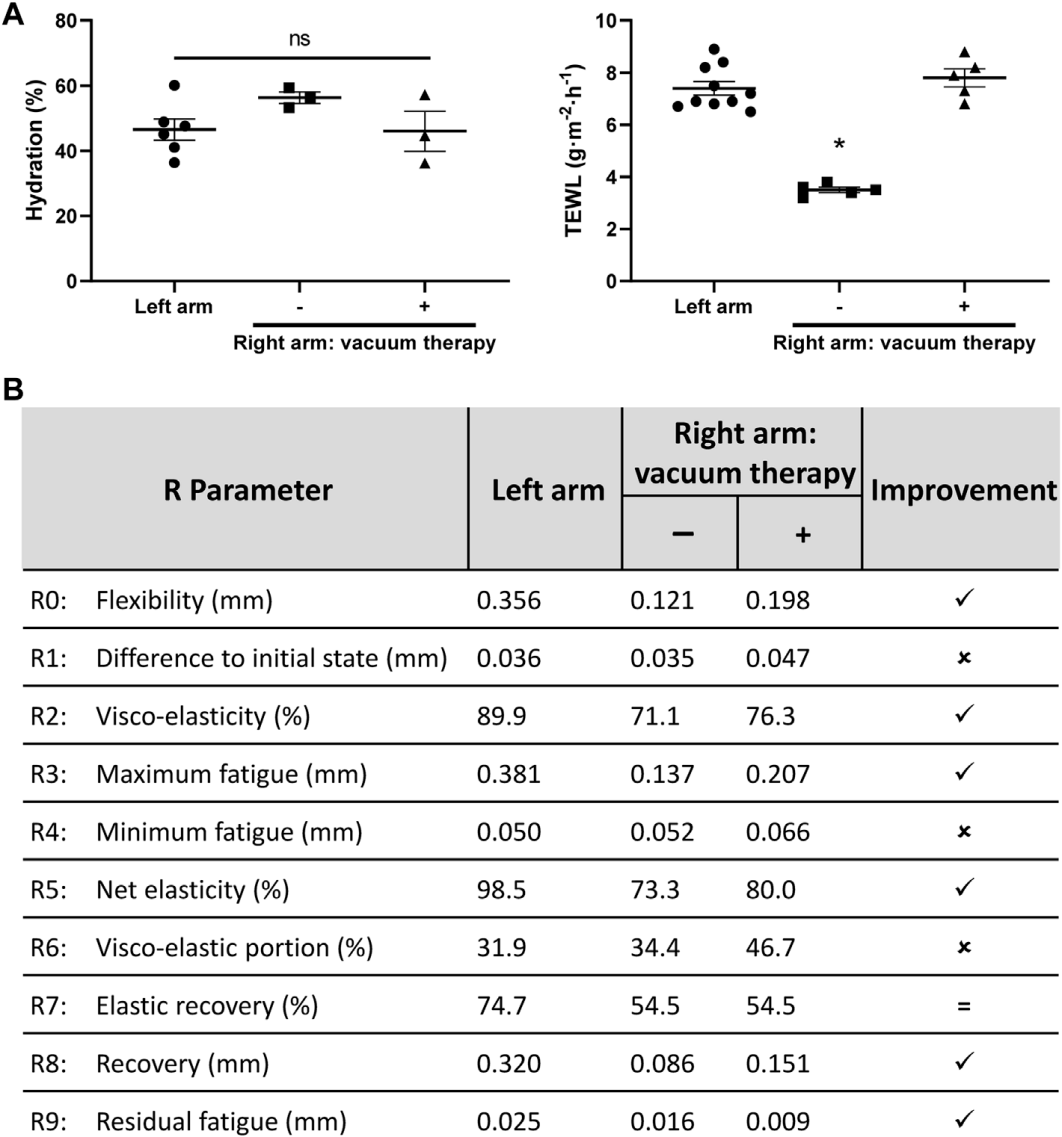
Mechanical and biophysical characterization of the skin in the regenerated area. **(A)** Hydration parameters of the skin. The hydration percentage, measured with a corneometer, is shown on the left, comparing both healthy skin from left arm with regenerated skin from right arm. The trans epidermal water loss (TEWL) is reported on the right, comparing both healthy skin from control arm with regenerated skin from right arm. Four measurements were made at either arm in each timepoint. The effect of the vacuum massage treatment on right arm can be seen for both parameters measured. Left arm values are the average of measurements at two different timepoints. ns, not significant differences; *p < 0.0001, one-way ANOVA. **(B)** Mechanical evaluation of the skin. Consecutive deformation cycles were performed on the skin using a cutometer, to assess for elastic properties of both healthy and regenerated skin. The measurements were repeated after the vacuum therapy. R parameters were calculated, and a qualitative improvement index is shown, stating if parameters improved on the right arm after the vacuum therapy sessions, relative to values obtained for the left arm.

Corneometry studies, performed to evaluate hydration of the skin, showed basal values of 46.5 ± 3.3% and 56.5 ± 1.8% for healthy and regenerated tissue respectively, which was not significantly affected by the vacuum massage sessions, maintaining a value of 46 ± 6.6% (Figure 3A, left). In contrast, trans epidermal water loss (TEWL) showed significant differences between the control and regenerated area, with basal values of 7.4 ± 0.26 g m^−2^∙h^−1^ and 3.5 ± 0.1 g m^−2^∙h^−1^ respectively, but such difference was reverted after vacuum massage therapy, reaching a value of 7.8 ± 0.35 g m^−2^∙h^−1^ (Figure 3A, right). Finally, sebum production was also measured and did not show significant differences between the analyzed tissues, with values​​ of zero obtained for all measurements (normal ranges <5 μg cm^−2^; data not shown). For corneometry and TEWL analyses, four measurements were made at either arm in each timepoint. Values for the left arm (control) corresponds to the average data of measurements performed at two time points in the same healthy arm (Figure 3A).

Skin flexibility on the regenerated tissue was measured using a Cutometer^®^ Dual MPA 580 device (Courage + Khazaka electronic GmbH, Köln, Germany), and compared to the control arm. The R parameters, calculated from the skin deflection data obtained with the suction cycles of the cutometer, are commonly reported in skin flexibility analysis and describe mechanical properties of the tissue (Woo et al., 2014; Sano et al., 2018; Courage+Khazaka Electronic GmbH, 2022b). Measurements revealed poor flexibility characteristics of the regenerated area, relative to the control arm (Figure 3B). This is mainly noted in parameters that account for flexibility, fatigue resistance and elasticity of the probed area (R0, R2, R3, R5 and R7), which deviate from values obtained in the control arm.

Notably, the skin flexibility increased after the vacuum massage therapy sessions in the regenerated area, as observed in the improvement of several R values. Flexibility (R0 value) increased from 0.121 mm to 0.198 mm (+63.6%) by the vacuum treatment. This improved value approaches the reference value of the control arm (0.356 mm) and is directly related with reduction in tissue fibrosis (Mueller et al., 2021). The treated area showed a conserved ability to return to its initial position after flexing, as R1 value was similar to control arm (0.047 vs. 0.036 mm). Maximum fatigue (R3) reached values closer to the initially measured R0, implying that skin mechanical fatigue was reduced in the regenerated area after the vacuum massage treatment. Values directly related to skin elasticity (R2, R5 and R7), also approached values closer to control after the vacuum therapy sessions, or maintained its value in the case of R7, emphasizing the mechanical improvement obtained with the directed treatment on the regenerated area (Figure 3B).

### Qualitative outcome and patient self-evaluation

**FIGURE 4:**
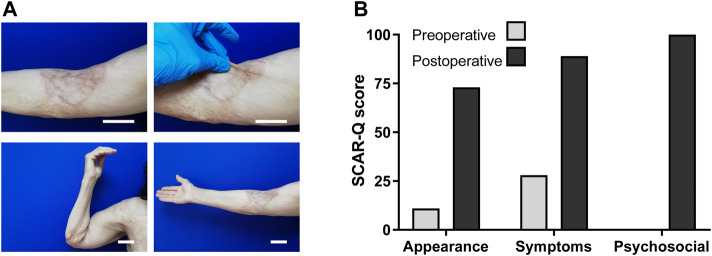
Qualitative outcome and self-evaluation. **(A)** Long-term photograph of the complete integration of photosynthetic scaffold and autologous split-thickness skin graft after 17 months. Detail of the skin graft outcome, and qualitative pinch-test performed in the treated area. Photographs show the entire arm achieving full-range flexion and extension, underscoring the gain in flexibility and elasticity in the regenerated area. Scale bars represent 5 cm. **(B)** A personal survey instrument to measure the patient-perceived outcome of the surgical procedure was applied at the month 17. The results reported in the three categories evaluated (appearance, symptoms and psychosocial) show an increased score in the self-perception of the injured area after the full treatment. An increased score in each category implies a better perception. No bar is observed in preoperative psychosocial survey, as SCAR-Q score was zero.

At month 17, a qualitative pinch test was performed on the regenerated skin, showing that the new tissue was flexible, allowing the patient to fully extend her arm (Figure 4A). In order to evaluate patient perception, a patient-reported outcome survey (SCAR-Q^©^) was used (Klassen et al., 2018). This self-reported instrument was designed for the assessment of surgical and traumatic scars, and evaluates patient perception regarding the appearance, symptoms, and psychological impact of the scar. The evaluation instrument was applied to the patient at the end of the follow-up study, to compare her perception about the arm before and after intervention. Values obtained from each of the three analyzed variables were transformed into a score ranging from 0 (worst) to 100 (best), using an equivalent Rasch transformed score (Klassen et al., 2018). Results shows a highly positive patient´s perception after the complete surgical and ambulatory procedures, i.e., 6.6-fold in appearance improvement, 3.1-fold in symptoms reduction and a response from 0 (worst) to 100 (best) score for the psychosocial perception (Figure 4B)**.**

## Discussion

The development of novel technologies to improve wound regeneration is an active field of research, with a high socioeconomic impact. Among the molecules involved in wound healing, oxygen has been broadly described to play a key role in almost every single step of the healing process (Tandara and Mustoe, 2004; Rodriguez et al., 2008). In fresh wounds, local oxygen tension is diminished due to the disruption of the microvascular network, creating an hypoxic environment that serves as the initial stimulus for the wound regeneration process. These hypoxic conditions increase keratinocyte motility, and induces HIF-1α expression, activating downstream genes related to glucose metabolism, erythropoiesis and angiogenesis (Rodriguez et al., 2008). Despite this, after these initial processes, oxygen presence is required for a proper wound healing process. Increased oxygen pressure is necessary for the adequate metabolism of the immune and epithelial cells involved in the regeneration process, and for the correct generation of oxygen-reactive species (ROS), which help signaling the immune system and combat potential bacterial infections. The presence of oxygen also aids in the collagen deposition, crucial for wound re-epithelization, and is fundamental for angiogenesis, which leads to the neovascularization of the regenerated tissue (Rodriguez et al., 2008; Lam Yip and Yip, 2015). In case of a failed wound oxygenation, a prolonged hypoxic microenvironment is associated among others with the development of tissue infections, and the establishment of a chronic wound (Tandara and Mustoe, 2004; Rodriguez et al., 2008; Eisenbud, 2012; Lam Yip and Yip, 2015; Younis, 2020). Therefore, novel strategies to increase local oxygen tension in wounds are required.

The patient described here is part of an ongoing clinical trial where oxygen-producing photosynthetic scaffolds are being implanted for the first time in human patients, representing the largest tissue defect treated in this cohort. After scar removal and photosynthetic scaffold implantation, an autograft was further implanted as part of the complete regeneration strategy. This second surgery is an standard procedure for covering and complementing the use of other commercially available scaffolds in different body locations such as scalp, limbs, and fingers (Azzena et al., 2010; Milcheski et al., 2014; Valerio et al., 2016; Chen and Song, 2017; De Haas et al., 2019; Zivec et al., 2022). Despite this, autograft implantation has never been described in a tissue substrate containing a large number of photosynthetic cells. In the patient presented here, it was clear that the presence of microalgae did not interfere with a long-term complete and stable tissue integration of the skin autograft. After 17 months of the first procedure, comprehensive characterization of the newly regenerated tissue reveals a proper functional outcome in terms of skin barrier as well as the mechanical properties of the tissue.

On her healthy control arm, the patient presented R parameters within the expected values when compared to age-matched individuals, especially for R2, R5 and R7 parameters, which on average were 0.64, 0.50 and 0.43 respectively (Gerhardt et al., 2009; Krueger et al., 2011; Kim et al., 2013).

Interestingly, after a vacuum massage treatment several key functional parameters also improved. The TEWL test, which is indicative of the integrity of the skin barrier, showing initial poor moisture permeability values in the regenerated skin, was restored to control levels. Similar results were observed for some mechanical parameters such as flexibility and elasticity, which reached almost normal skin features. It is worth noting that, although the vacuum massage therapy is normally used as an aesthetic procedure, a relevant clinical improvement was achieved in this case, broadening the margin of further improvement in this type of regenerated tissues. This is possibly due to the stimulation of blood circulation and cyclical stretching of the skin caused by the vacuum massage therapy, which might have enhanced the tissue regeneration in the treated area. However, further studies in a larger cohort have to be performed in order to confirm the effectiveness of the local vacuum therapy in the improvement of the functional and mechanical characteristics of the skin treated with our photosynthetic scaffold.

From the functional point of view, i.e., elbow flexo-extension, the patient was able to fully extend her arm for the first time since childhood. This proves the fact that in this particular patient, the presence of photosynthetic microalgae in the scaffold did not impair the long-term process of tissue regeneration, integration, remodeling, and functional restoring of the defect, improving the patient’s self-perception related to the injury and alleviating the psychosocial burden that arose from the presence of the burn scar.

The photosynthetic approach described here is still strongly limited by the need to develop proper and effective illumination devices for wounds as well as for all the other potential applications of this technology. Moreover, to date no data has been reported showing efficacy of photosynthetic therapy in humans, and therefore clinical data is urgently required in order to fully assess the potential therapeutic effect of photosynthetic cell implantation. Nevertheless, the data presented here represents an important step towards the goal of therapeutic photosynthesis, as it shows the feasibility of this concept in a long-term study where auspicious local and functional outcomes were observed.

## Conclusion

The follow-up case report presented here, shows that no detrimental consequences nor local or systemic side effects can be observed after 17 months of treating a large full-thickness skin defect with a photosynthetic scaffold. In fact, a significant improvement in the quality of life of the patient was evidenced, as the patient recovered the ability to fully extend and flex the elbow. Moreover, vacuum massage treatment improved the quality of the regenerated tissue, therefore enhancing the successful outcome of this procedure. Altogether, this clinical case report confirms the long-term safety of implanting photosynthetic microorganisms in human patients.

## Data Availability

The raw data supporting the conclusions of this article will be made available by the authors, without undue reservation.

## Supplementary material

Supplementary Figure S1 Overview of the photosynthetic scaffold fabrication and implementation on the wound. Microalgae were resuspended in fibrin and seeded on Integra® dermal regeneration template. Scaffolds were incubated for 4 days under constant illumination while microbiology testing was performed. Surgical implantation in the wound was performed, and the scaffolds were illuminated for 7 days to stimulate photosynthesis. Following adequate tissue integration, autografting surgery was performed after 21 days of photosynthetic scaffold implantation.

Supplementary Figure S2 Post-surgical self-evaluation questionnaire. The patient was evaluated daily for 10 days after the photosynthetic scaffold implantation surgery in terms of the self-perception score of five parameters: perceived pain **(A)**, ranging from 0 to 10, itching **(B)**, ranging from 0 to 3, burning sensation **(C)**, ranging from 0 to 3, smell associated with the implanted photosynthetic scaffold **(D)**, ranging from 0 to 3, and light annoyance related to the illumination device **(E)**, ranging from 0 to 3. Illumination device was removed from the patient after day 7. Data extracted from (Obaíd et al., 2021).
