# Supplementary material for: Case report: Long-term follow-up of a large full-thickness skin defect treated with a photosynthetic scaffold for dermal regeneration
Source: Front Bioeng Biotechnol. 2022 Dec 1;10:1004155. doi: 10.3389/fbioe.2022.1004155 (PMC9751053; doi:10.3389/fbioe.2022.1004155)
Supplement: Supplementary file 1 [file DataSheet1.pdf]

frontiers

Supplementary figures for:

Case Report: Long-Term Follow-up of a Large Full-Thickness Skin Defect Treated with a Photosynthetic Scaffold for Dermal Regeneration

Miguel L. Obaíd[1†], Felipe Carvajal[2†], Juan P. Camacho[1], Rocío Corrales-Orovio[2,3], Ximena Martorell[4], Juan Varas[5], Wilfredo Calderón[1], Christian Dani Guzmán[6], Marianne Brenet[2], Margarita Castro[8], Cecilia Orlandi[8], Sebastián San Martín[5], Antonio Eblen-Zajjur [2,7], José T. Egaña[2]*.

[1]Department of Plastic Surgery, Hospital del Salvador, Santiago, Chile.

[2]Institute for Biological and Medical Engineering, Schools of Engineering, Medicine and Biological Sciences, Pontificia Universidad Católica de Chile, Santiago, Chile.

[3]Division of Hand, Plastic and Aesthetic Surgery, University Hospital, LMU, Munich, Germany.

[4]Critical Care Unit, Hospital del Salvador, Santiago, Chile.

[5]Biomedical Research Center, School of Medicine, Universidad de Valparaíso, Valparaíso, Chile.

[6]Sky-Walkers SpA, Litueche, Chile.

[7]Translational Neuroscience Lab, Faculty of Medicine, Universidad Diego Portales, Santiago, Chile.
[8]Clínica Orlandi Dermatological Center, Santiago, Chile.

[†]These authors have contributed equally to this work and share first authorship

*** Correspondence:**

José T. Egaña

jte@uc.cl

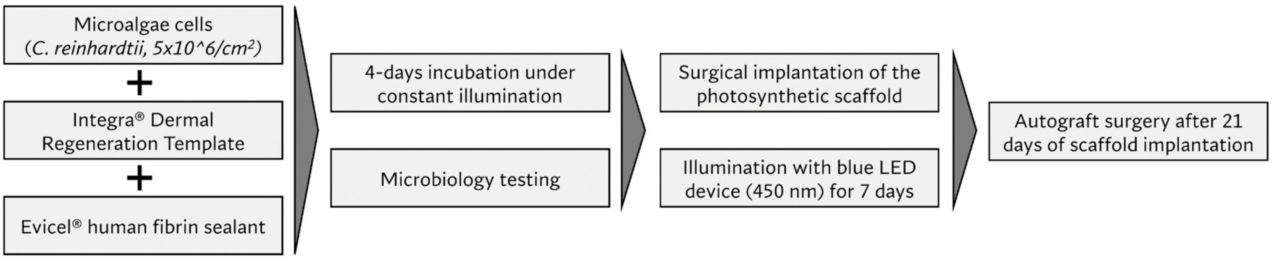

Supplementary figure 1: Overview of the photosynthetic scaffold fabrication and implementation on the wound. Microalgae were resuspended in fibrin and seeded on Integra® dermal regeneration template. Scaffolds were incubated for 4 days under constant illumination while microbiology testing was performed. Surgical implantation in the wound was performed, and the scaffolds were illuminated for 7 days to stimulate photosynthesis. Following adequate tissue integration, autografting surgery was performed after 21 days of photosynthetic scaffold implantation.

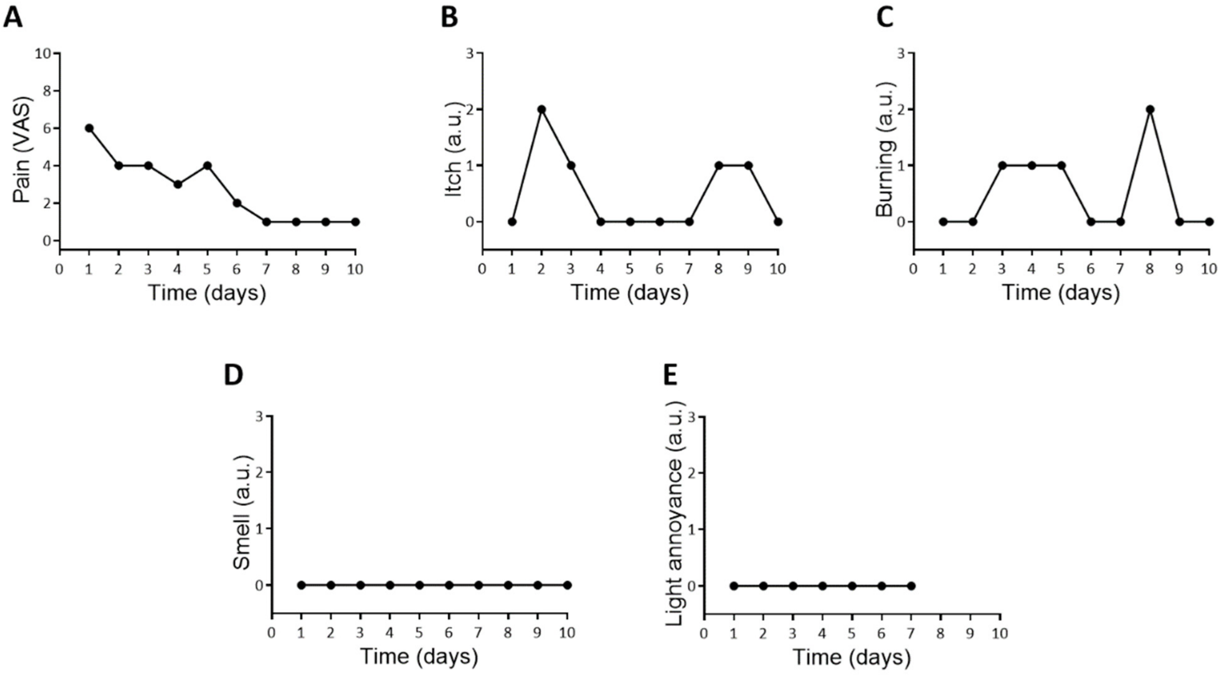

Supplementary figure 2: Post-surgical self-evaluation questionnaire. The patient was evaluated daily for 10 days after the photosynthetic scaffold implantation surgery in terms of the self-perception score of five parameters: perceived pain (**A**), ranging from 0 to 10, itching (**B**), ranging from 0 to 3, burning sensation (**C**), ranging from 0 to 3, smell associated with the implanted photosynthetic scaffold (**D**), ranging from 0 to 3, and light annoyance related to the illumination device (**E**), ranging from 0 to 3. Illumination device was removed from the patient after day 7. VAS, visual analogue scale. a.u.: arbitrary units. Data extracted from (Obaíd et al., 2021).
